# Supplementary material for: Structural basis for synthase activation and cellulose modification in the E. coli Type II Bcs secretion system
Source: Nat Commun. 2024 Oct 11;15:8799. doi: 10.1038/s41467-024-53113-8 (PMC11470070; doi:10.1038/s41467-024-53113-8)
Supplement: Supplementary file 1 — Supplementary Information [file 41467_2024_53113_MOESM1_ESM.pdf]

Supplementary Information

for

Structural basis for synthase activation and cellulose modification in the *E. coli* Type II Bcs secretion system

Itxaso ANSO[1,2,3], Samira ZOUHIR[1,2,4] ,Thibault G. SANA[1,2] and Petya Violinova KRASTEVA[1,2,*]

[1] Univ. Bordeaux, CNRS, Bordeaux INP, CBMN, UMR 5248, F-33600 Pessac, France

[2] 'Structural Biology of Biofilms' Group, European Institute of Chemistry and Biology (IECB), 2 Rue Robert Escarpit, Pessac F-33600, France

[3] Department of Biochemistry and Molecular Biology, Faculty of Science and Technology, University of the Basque Country (UPV/EHU), Barrio Sarriena s/n, 48940 Leioa, Spain

[4] Current address: Laboratoire de Biologie et Pharmacologie Appliquée (LBPA), CNRS UMR8113, ENS Paris-Saclay, Université Paris-Saclay, Gif-sur-Yvette F-91190, France

* Correspondence: pv.krasteva@iecb.u-bordeaux.fr

This document includes:

Supplementary Fig. 1-9

Supplementary Tables S1-S4

Supplementary References

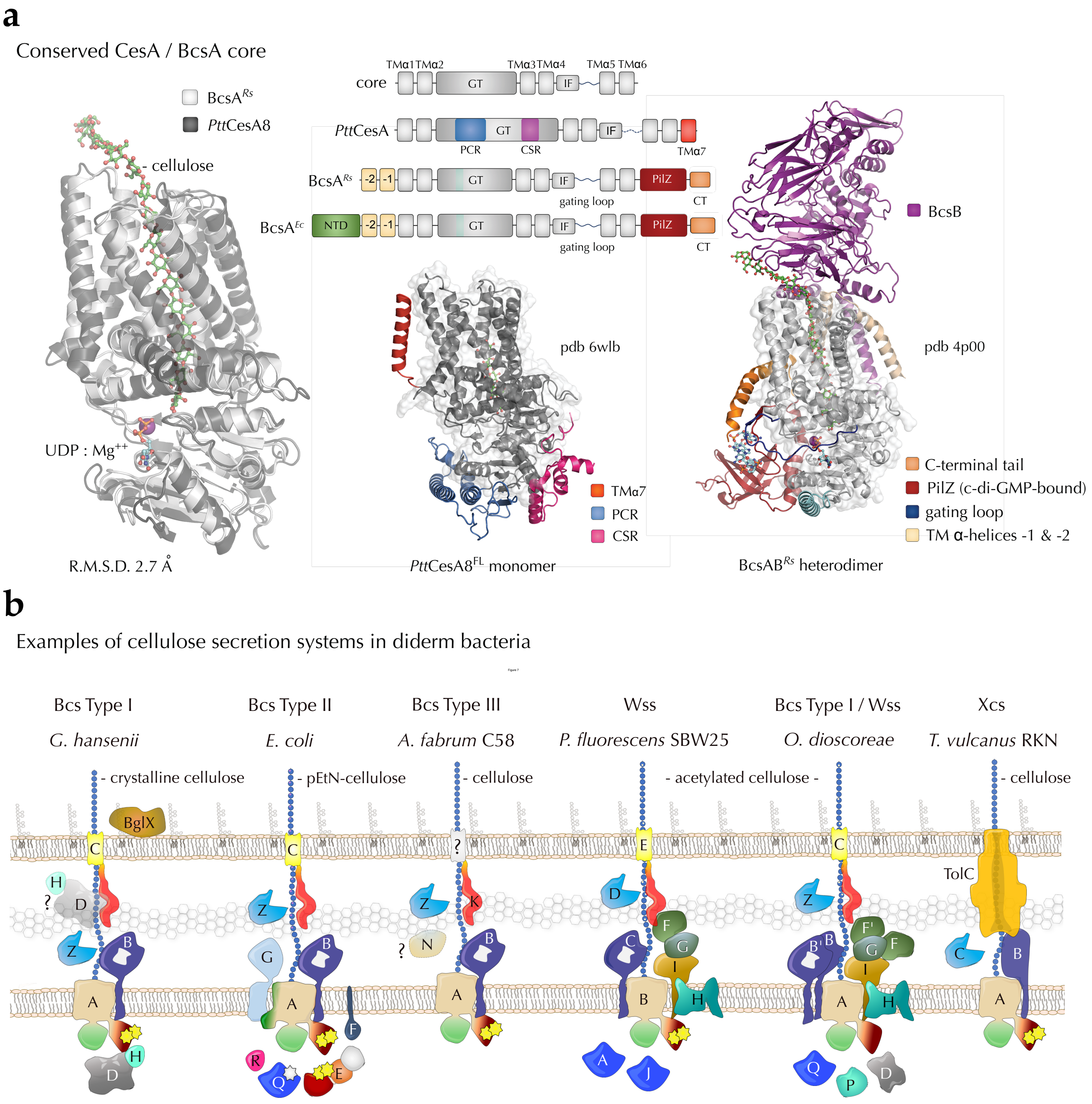

Supplementary Fig. 1 Synthase conservation vs. variety of Bcs secretion systems. a Conservation of the core synthase fold between bacterial (BcsA) and plant (CesA) cellulose synthases. *Rs*, *Rhodobacter sphaeroides*; *Ptt*, *Populus tremula* × *tremuloides* or poplar; *Ec*, *E. coli;* UDP, uridine diphosphate; R.M.S.D., root mean square deviation; TMα, transmembrane helix; IF, interface helix; PCR, plant-conserved region; CSR, class-specific region; FL, full-length. Adapted with modifications from Abidi *et al.* 2022[1] under Creative Commons-NonCommercial License CC BY-NC 4 (http://creativecommons.org/licenses/by-nc/4.0/) **b** Examples of Bcs secretion systems across diderm bacteria. Adapted with modifications from Krasteva 2024[2] under the CC BY 4.0 license (https://creativecommons.org/licenses/by/4.0/legalcode).

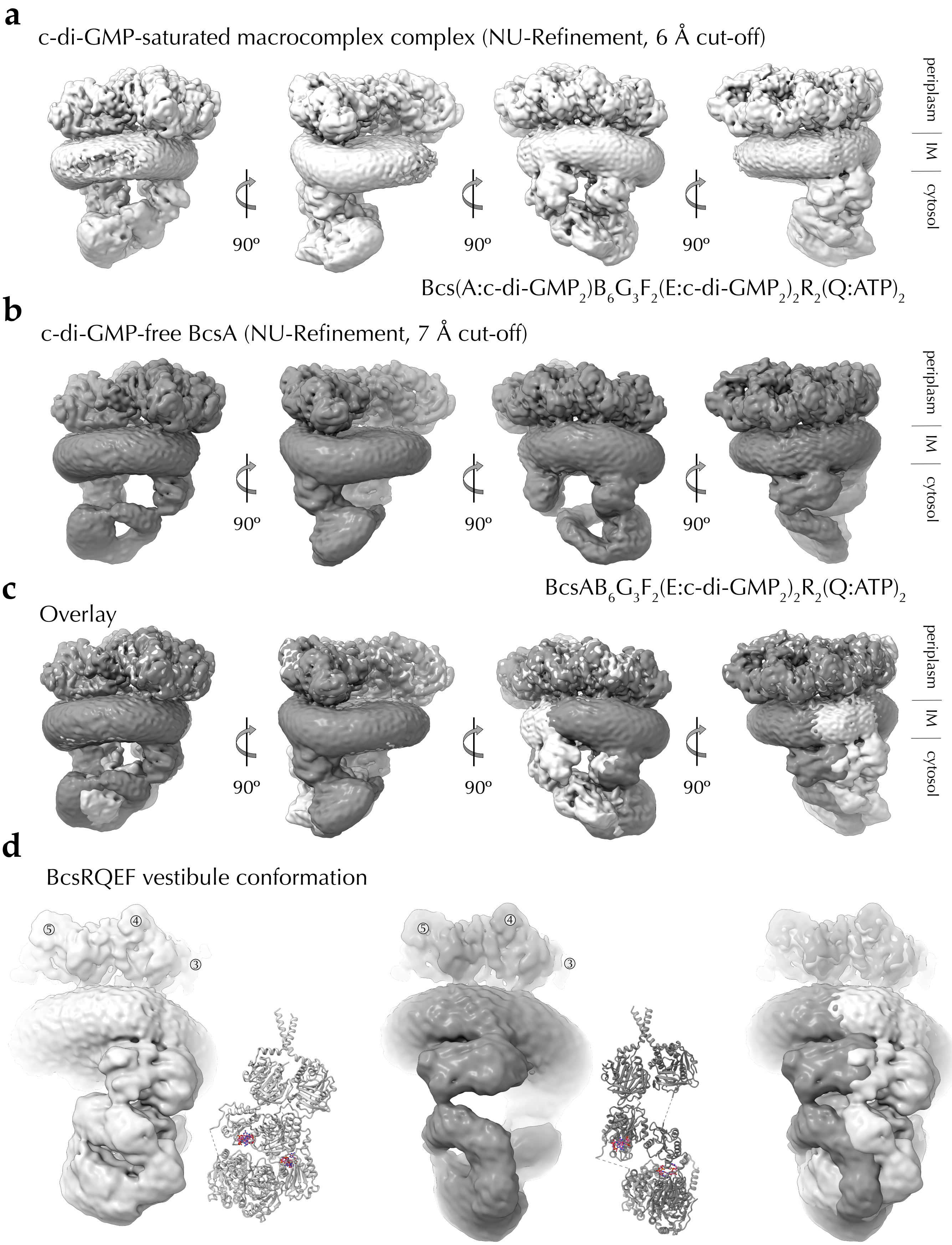

Supplementary Fig. 2 Global architecture of the *E. coli* Bcs macrocomplex. a-b The two captured states, featuring a c-di-GMP-bound **(a)** or a c-di-GMP-free **(b)** BcsA synthase are shown as electron density map reconstructions refined to 6 Å and 7 Å, respectively. NU-refinement, non-uniform refinement **c** Overlay of the two resolved states. **d** Close-ups of the vestibule regions corresponding to the BcsRQEF regulators shown in the corresponding maps and in cartoon representations with the BcsE-bound c-di-GMP dimers in sticks. Left, GMP-saturated state; middle, subcomplex accompanying the c-di-GMP-free synthase; right, map overlay.

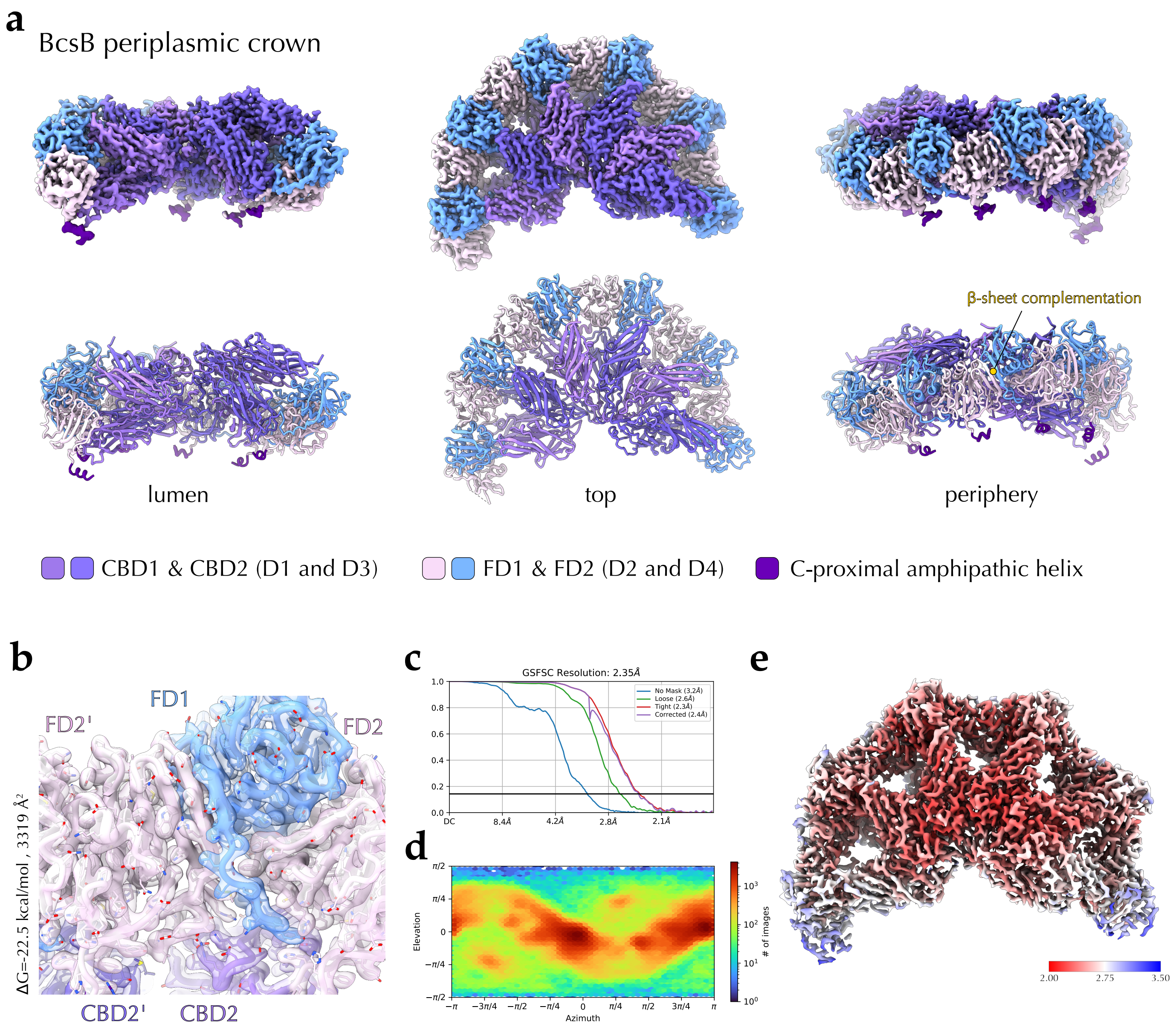

Supplementary Fig. 3 Cryo-EM structure of the *E. coli* BcsB periplasmic crown. a Cryo-EM density map and atomic model (in cartoon). D, domain; CBD, carbohydrate-binding domain; FD, flavodoxin-like domain. **b** A close-up of the model-in-map at the interprotomer interface. Free energy gain and buried surface area calculated by the PISA server. **c** Gold-standard Fourier shell correlation (GSFSC) curve used to determine the average density map resolution (cryoSPARC[3]). **d** Viewing direction distribution across the dataset (cryoSPARC[3]). **e** Surface mapping of the local resolution (2-3.50 Å in a red-blue gradient).

a

Non-saturated state (c-di-GMP-free synthase; Local Refinement)

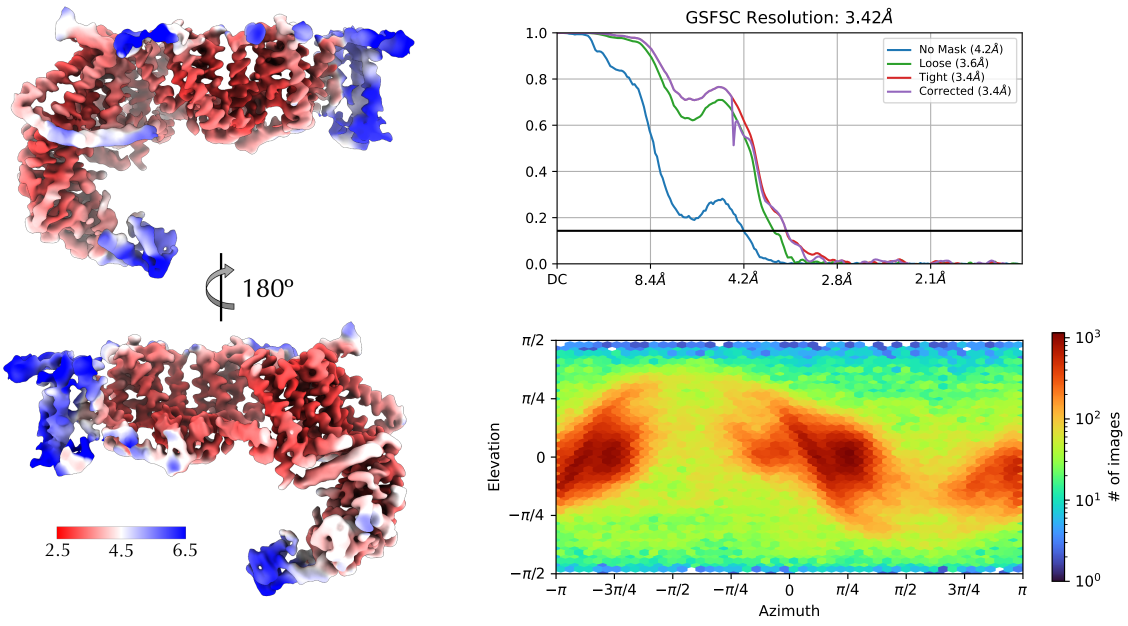

b

$BcsAG_3$ in the c-di-GMP-saturated Bcs macrocomplex (Local Refinement)

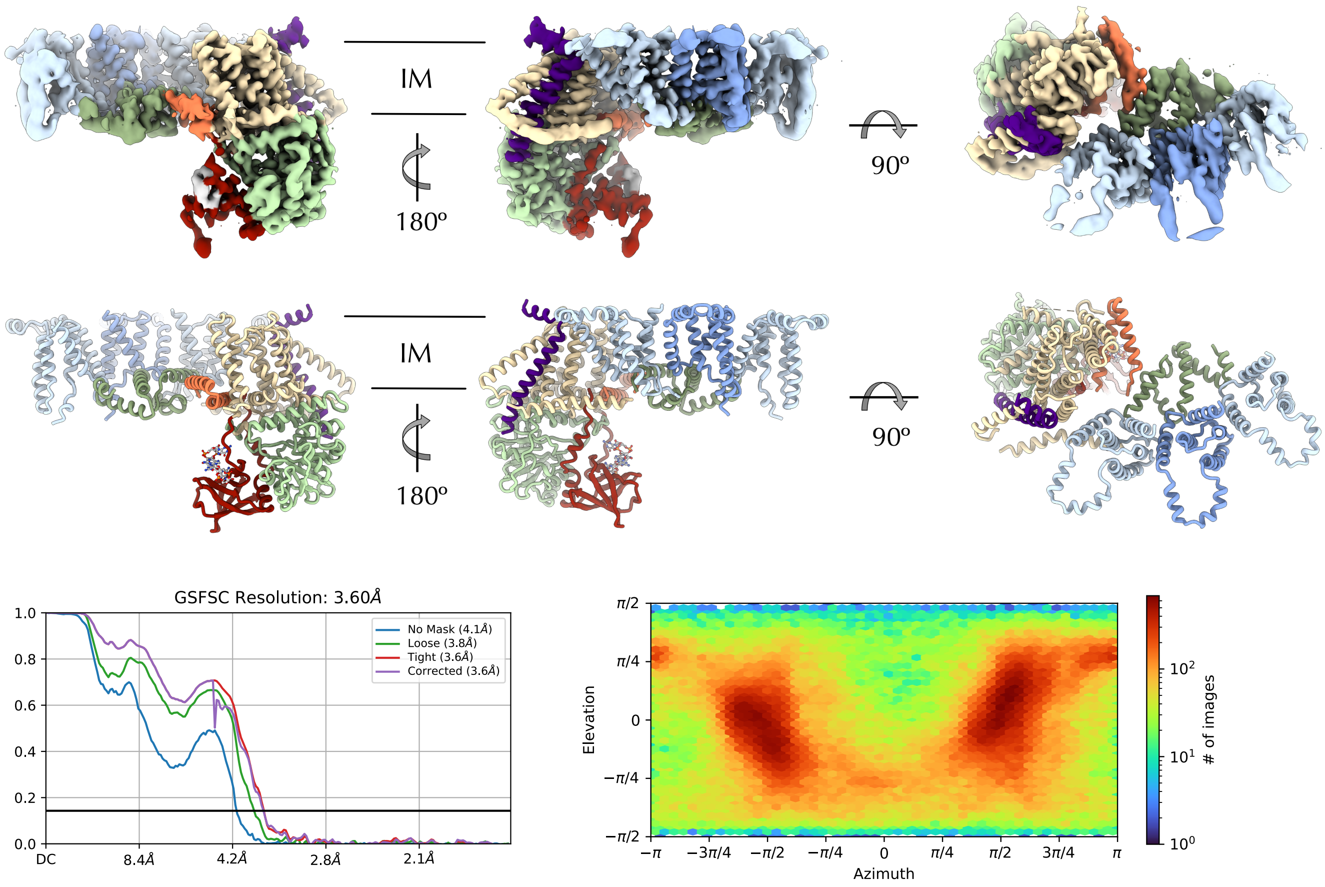

Supplementary Fig. 4 Cryo-EM structures of the $BcsAG_3$ assemblies : supplementary information. a Left, local resolution mapping as a red-blue color gradient onto the sharpened cryo-EM map of the c-di-GMP-free $BcsAG_3$ assembly. Right, the GSFSC (Gold-Standard Fourier shell correlation curves) and viewing direction distribution across the dataset. **b** Cryo-EM structure of the c-di-GMP-bound $BcsAG_3$ complex shown as a locally refined electron density map and an atomic model in cartoon. Bottom, GSFSC curve and viewing direction distribution.

a

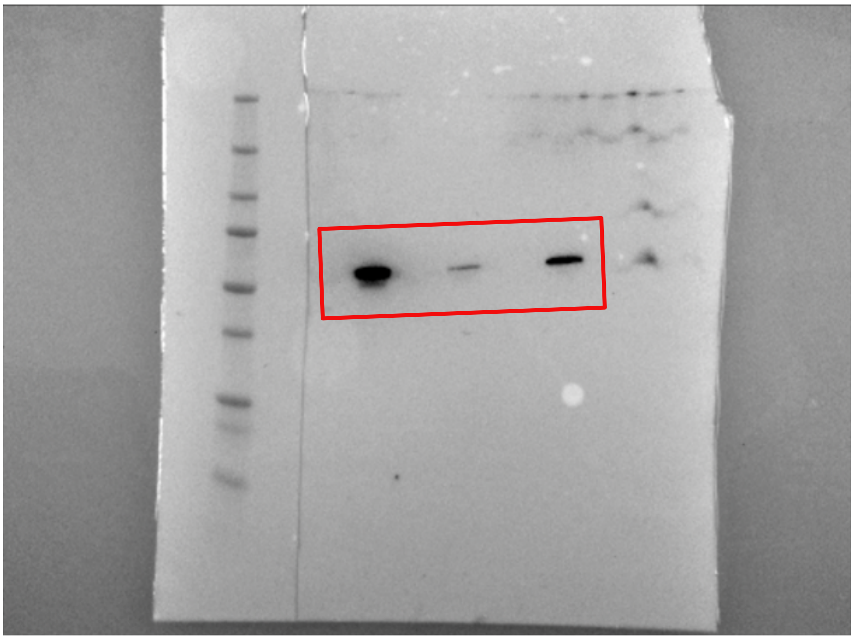

b

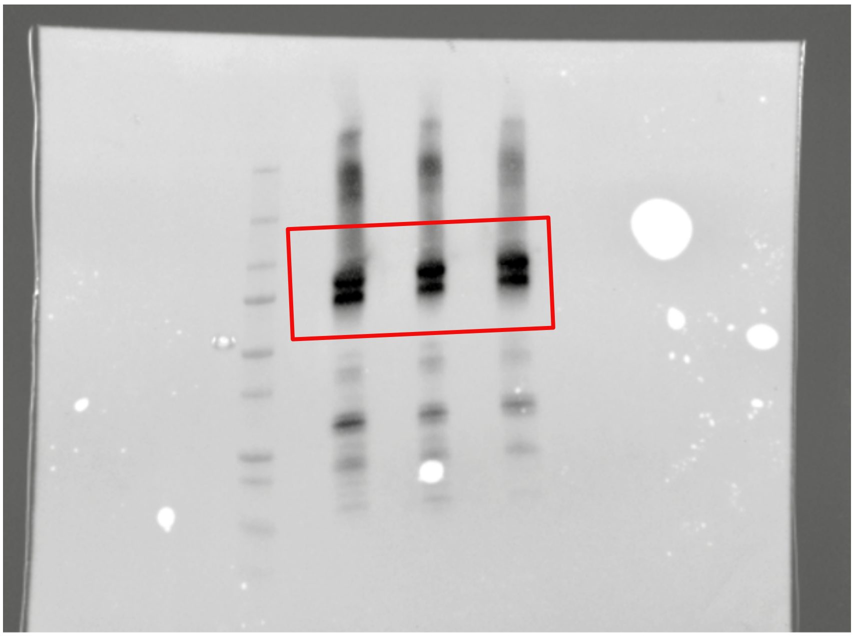

Supplementary Fig. 5 Uncropped blots (partially visualized in Fig. 3c) a Primary mouse anti-STREP II antibody QIAGEN, #34850; dilution 1:1000); secondary horseradish peroxidase (HRP)-conjugated rabbit anti-mouse antibody (Abcam, ab6728; dilution 1:10,000). Due to strong signal from the Bio-Rad Precision Protein Plus ladder, it was excised prior to antibody incubation and added for the signal visualization. **b** Primary mouse anti-HA (hemagglutinin) (Thermo Fisher Scientific, #26183; dilution 1:1000); secondary antibody as in **(a)**.

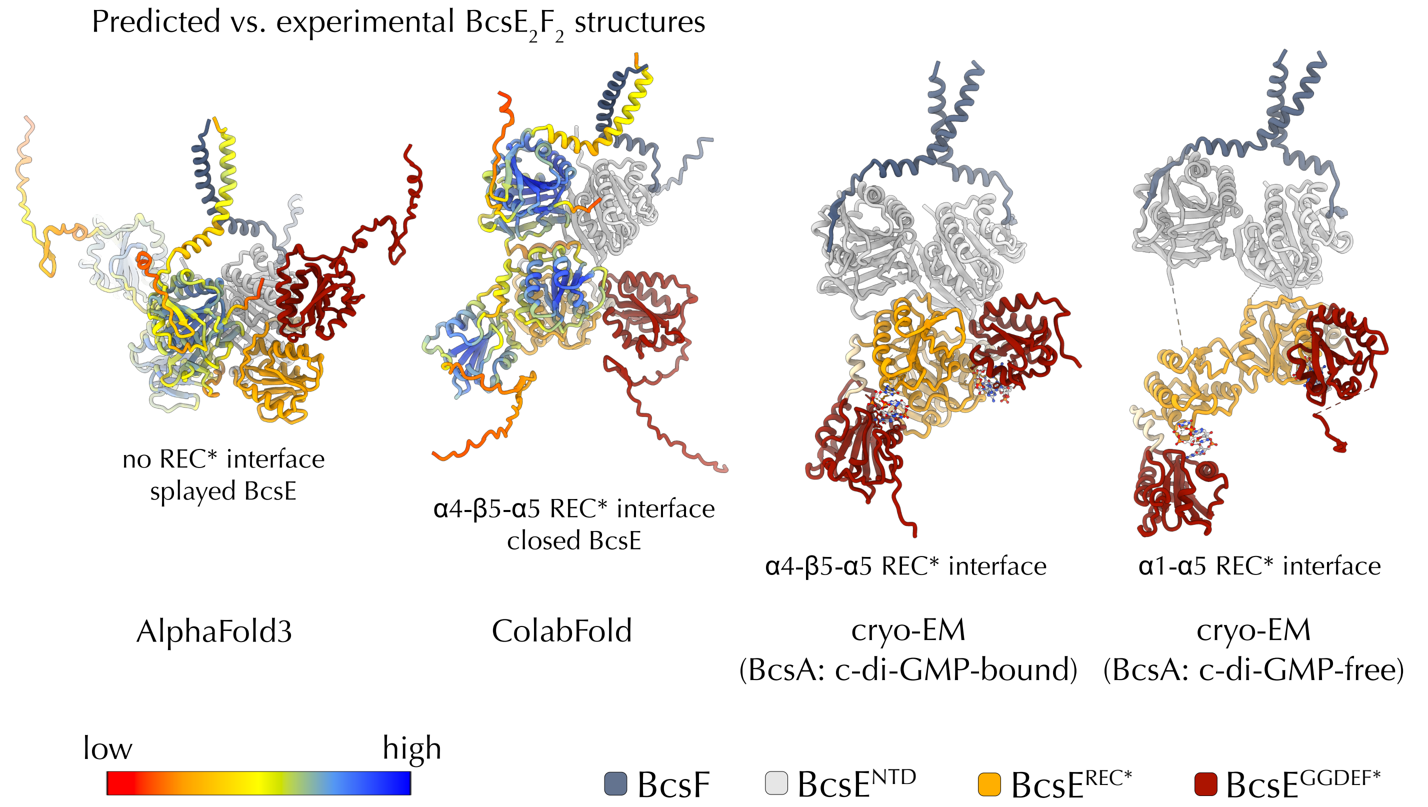

Supplementary Fig. 6 Predicted vs. experimental structures of enterobacterial E_2F_2 regulatory complex. From left to right: an AlphaFold 3[4]-based model using consensus BcsE and BcsF sequences from representative cellulose-secreting enterobacteria; a ColabFold[5]-predicted model using the same input sequences; cryo-EM structure of BcsEF in the c-di-GMP-saturated Bcs macrocomplex from *E. coli* (this study); cryo-EM structure of the same assembly in complex with a c-di-GMP-free BcsE (this study). Whereas head-to-head $BcsE^{NTD}$ dimerization and peripheral β-sheet complementation by BcsF are present in both predicted models, the experimental structures reveal additional conformational space for the BcsE cellulose secretion enhancer.

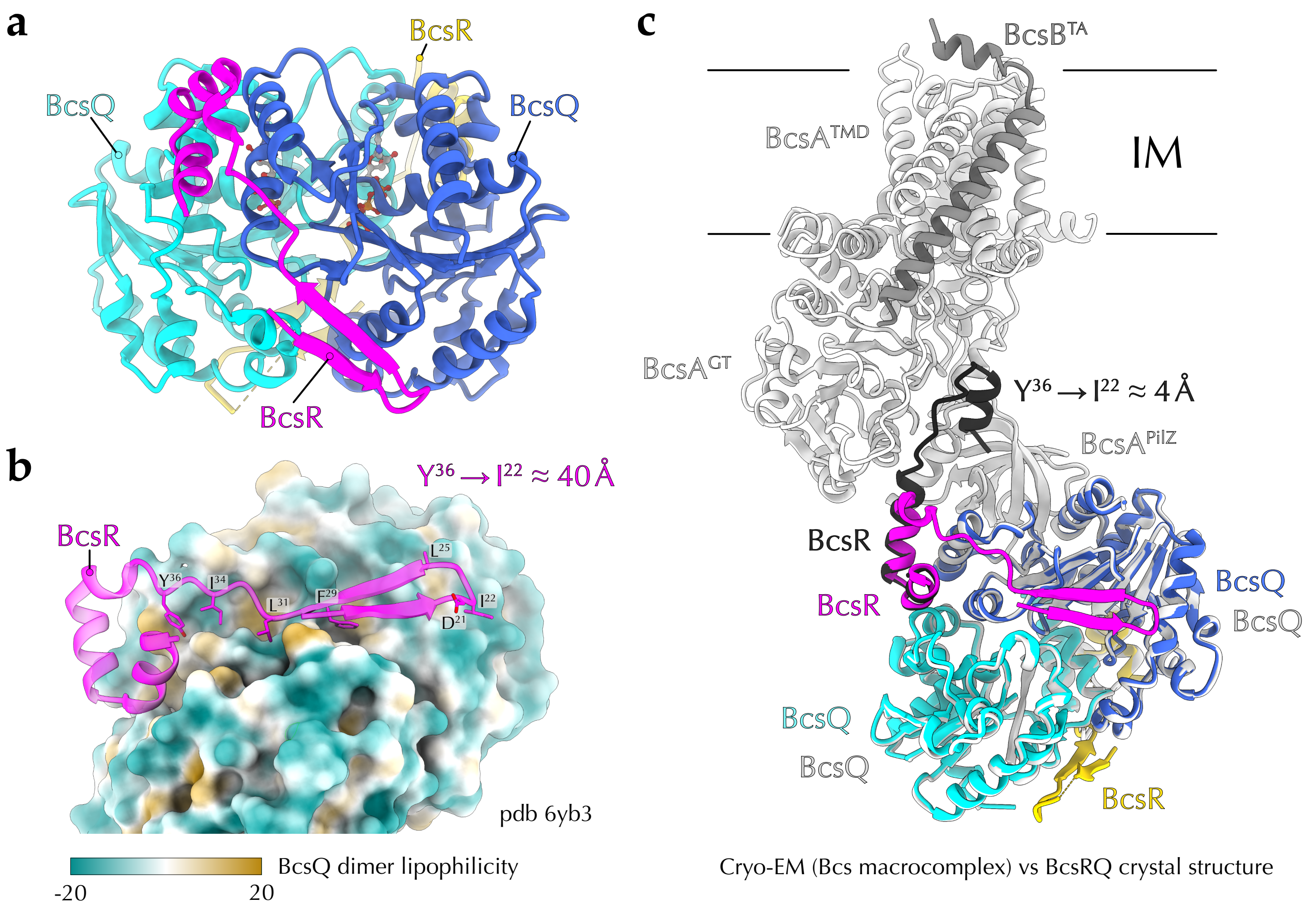

Supplementary Fig. 7 BcsR conformations in the BcsRQ complex (crystal structure) vs. the Bcs macrocomplex (cryo-EM) **a** Crystal structure of the BcsRQ crystal structure (pdb: 6yb3)[6] with BcsR protomers colored in magenta and gold and BcsQ copies colored in cyan and blue). The N-terminal BcsR domain adopts a β-hairpin fold. **b** Cartoon vs. lipophilicity-colored surface representation of a BcsR protomer on the surface of the BcsRQ dimer. Key BcsR amino acids participating in BcsR-BcsA interactions are shown as sticks. I^{22} and Y^{36} are positioned more than 40 Å apart. **c** Overlay of the crystal (colors as in **a**) and cryo-EM (in shades of grey) structures highlighting the difference in BcsR conformation. The side chains of I^{22} and Y^{36} are less than 4 Å apart. For simplicity, only the core TMD-GT-PilZ domains of BcsA, the tail anchor of interacting BcsB and the BcsRQ subunits are shown.

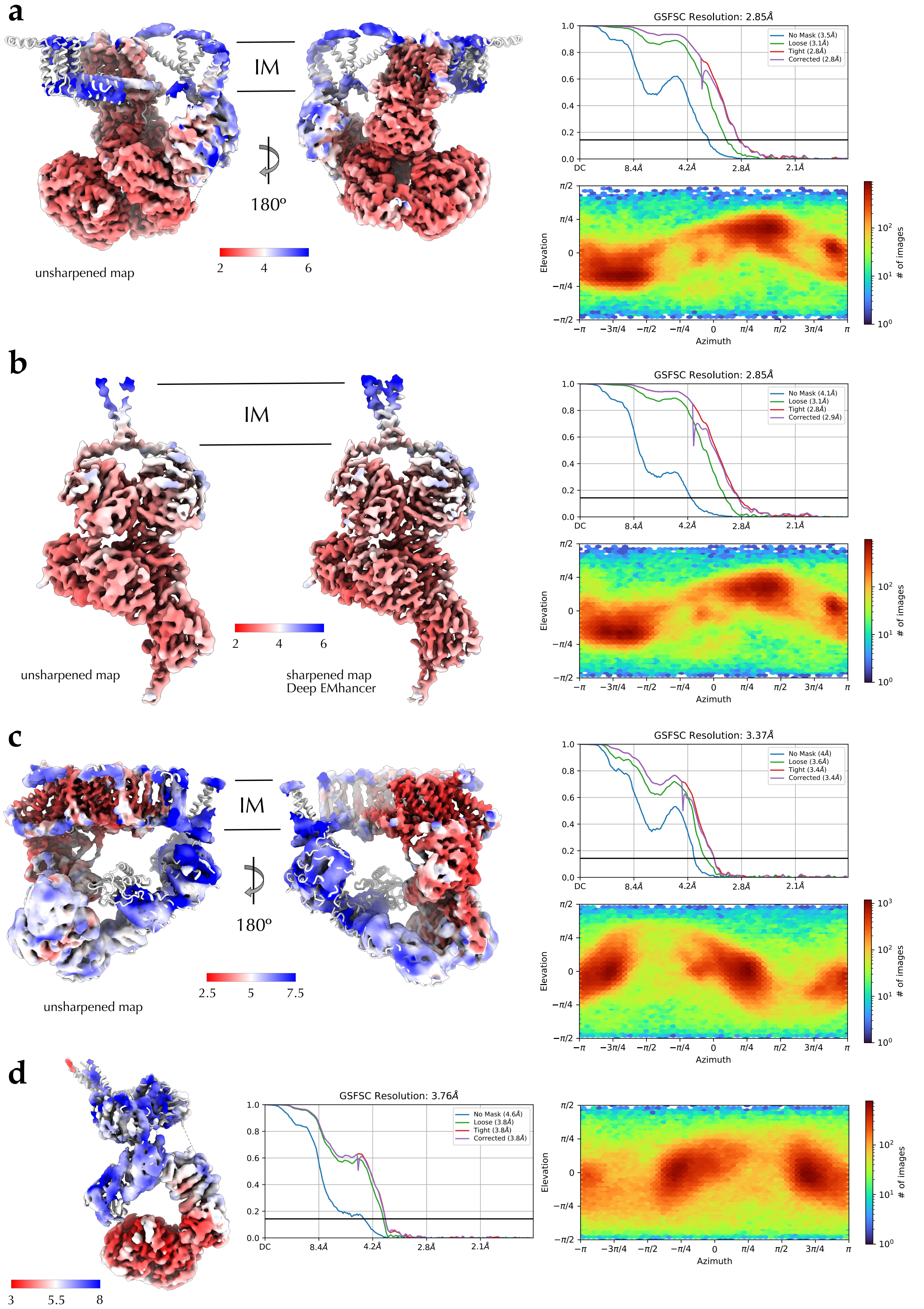

Supplementary Fig. 8 Cryo-EM structures of the crownless Bcs macrocomplexes and corresponding locally refined vestibule subcomplexes, supplementary information. a-b c-di-GMP saturated complex; **c-d** non-saturated complex. Local resolution surface mapping (in Å) on the unsharpened and/or Deep EMhancer[7]-sharpened maps, GSFSC curves and viewing direction distributions are shown for each locally refined assembly (cryoSPARC[3]).

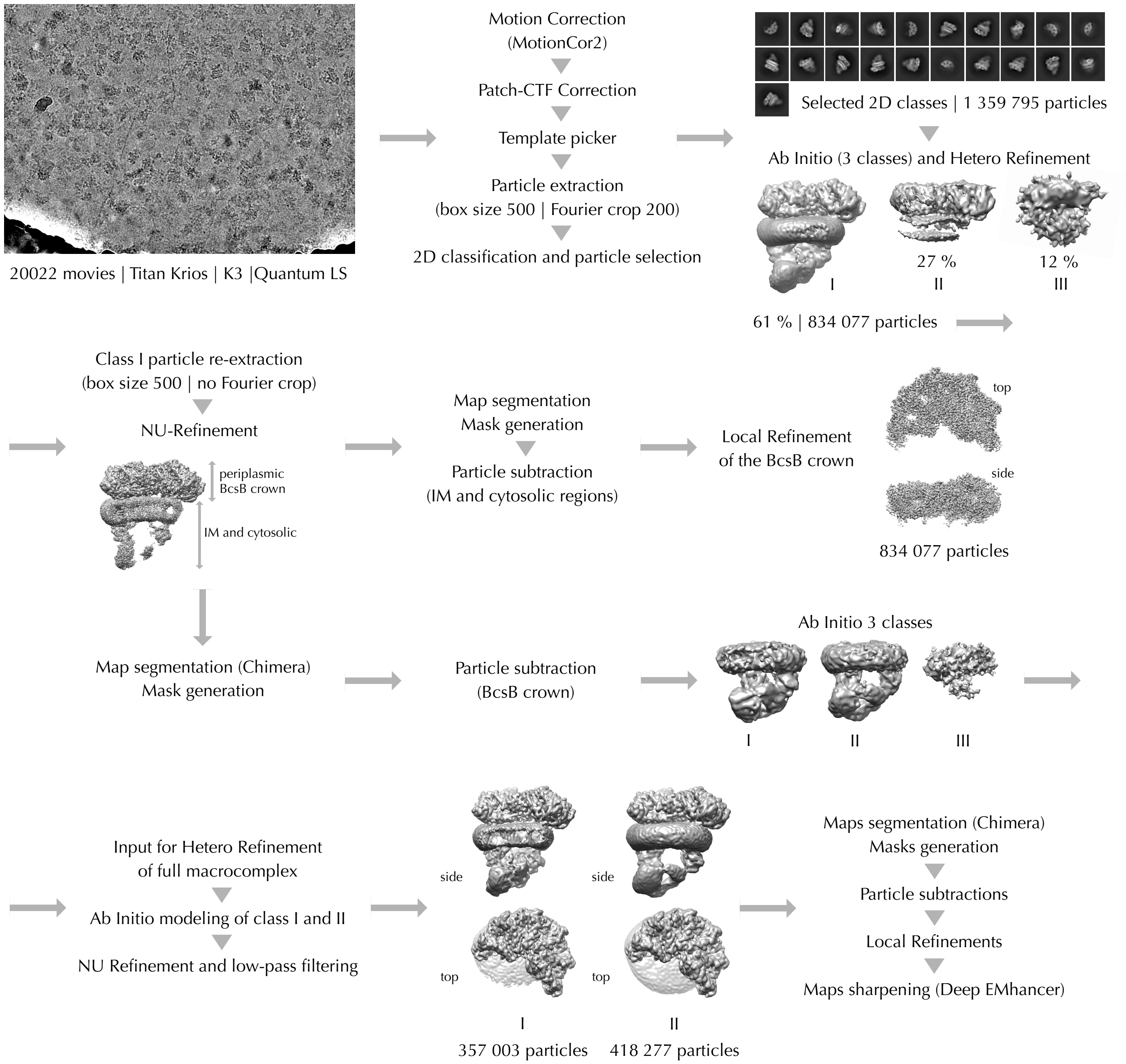

Supplementary Fig. 9 Details on the cryo-EM data processing workflow. All processing was done in cryoSPARC[3] v4.4.1 with the exception of Motion Correction (MotionCor2[4]) and map segmentations for local refinements (Chimera[5]). Maps sharpening for density interpretation of lower-resolution regions was carried out with Deep EMhancer[6] through the cryoSPARC interface. Data collection was performed on a Titan Krios, 300 kV transmission cryo-electron microscope (CM01 at the European Synchrotron Radiation Facility; Thermo Fisher Scientific); GATAN K3, direct electron detector; Quantum LS, energy filter (GATAN).

Table S1 | Oligonucleotides and construct design

Oligonucleotide	Sequence	Purpose
Expression constructs		
pACYC_s1_His_Pst_as	TCGATC**CTGCAG**C**GTGATGGTGGTGATGATGGTGATG**GCT GCTGCC**CAT**G	pACYCDuet1 amplification for NotI/PstI-based cloning in MCS1and His_8-tagged protein expression
pACYC_s1_Not_s	CTCGAG**GCGGCCGC**ATAATGCTTAAGTCGAACAGA	pACYCDuet1 amplification for NotI/PstI-based cloning in MCS1and His_8-tagged protein expression
BcsR_1_His_Pst_s	CATCACG**CTGCAG**GATCGAATAACAATGAACCAGATACTC	Used in restriction-based cloning of *E. coli* $bcsRQA^{HA\text{-}FLAG}B$ in MCS1 of pACYCDuet1 to yield pACYC-$bcs^{His}RQA^{HA\text{-}FLAG}B$ (BcsR: M^1GSSHHHHHHHHAAGSN^{2}N...)
BcsB_779_Not_as	CTAGAA**GCGGCCGCTTA**CTCGTTATCCGGGTTAAGACGA CGACGAC	Used in restriction-based cloning of *E. coli* $bcsRQA^{HA\text{-}FLAG}B$ in pACYCDuet1 to yield pACYC-$bcs^{His}RQA^{HA\text{-}FLAG}B$
BcsF_ΔNTD_s	GCTAAACCTCGTTATGTTAAACCGGCCG	Used in inverse PCR to delete the N-terminal region of BcsF in the pRSFDuet1-^{Strep}EFG expression construct (M^1M-A^{41}KPRYVKPA...)
BcsF_ΔNTD_as	CATCATGATGAGCGCTCCACAGCATCATCTAAC	Used in inverse PCR to delete the N-terminal region of BcsF in pRSFDuet1-^{Strep}EFG
BcsF_ΔCTD_s	CT**TAAT**CGTTATGTTAAACCGGCCGGGACGTTAC	Used in inverse PCR to insert a four-letter TAAT stop codon in BcsF after P^{43} in pRSFDuet1-^{Strep}EFG
BcsF_ΔCTD_as	GTTTAGCAAAGAACAAACGTAAGGTGTCGCGAATG	Used in inverse PCR to insert a four-letter TAAT stop codon in BcsF after P^{43} in pRSFDuet1-^{Strep}EFG
BcsR_D21K_pAM_s	**G**ATTGTGGCCGTTAAAGCAGGCATTTTCACTGCC	Used in inverse PCR to introduce a D^{21}K point mutation in pAM238-cloned *bcsR*
BcsR_D21K_pAM_as	**TT**ATTCTGGAAGATATAGCCTATCGCGGGATCAGGC	Used in inverse PCR to introduce a D^{21}K point mutation in pAM238-cloned *bcsR*
BcsR_KDDD_pAM_s	CAGGCA**GAT**TCA**GAT**CCTGATATTGATTATGCCGATA TTTCCCAACG	Used in inverse PCR to introduce D^{21}K-L^{25}D-F^{29}D-L^{31}D point mutations in pAM238-cloned *bcsR*
BcsR_KDDD_pAM_as	CTT**ATC**CGCCACAAT**CTT**ATTCTGGAAGATATAGCCTATC GCGG	Used in inverse PCR to introduce D^{21}K-L^{25}D-F^{29}D-L^{31}D point mutations in pAM238-cloned *bcsR*
BcsR_ADDDA_pAM_s	CAGGCA**GAT**TCA**GAT**CCTGATATTGAT**GCT**GCCGATATT TCCCAACGC	Used in inverse PCR to introduce D^{21}A-L^{25}D-F^{29}D-L^{31}D-Y^{36}A point mutations in pAM238-cloned *bcsR*
BcsR_ADDDA_pAM_as	CTT**ATC**CGCCACAAT**CGC**ATTCTGGAAGATATAGCCTAT CGCG	Used in inverse PCR to introduce D^{21}A-L^{25}D-F^{29}D-L^{31}D-Y^{36}A point mutations in pAM238-cloned *bcsR*
Chromosomal mutants		
KanCst_pKD4_up_s-bcs2	CGCCCGGACCAGGCCGCAGGGGGGAAACTCTGCGG CCTTTTTCGTTCTTATGTGTAGGCTGGAGCTGCTTCG	Used for amplification of the FRT-flanked Km^R cassette from pKD4 with ~50 bp overhangs homologous to the *bcs*-flanking regions
KanCst_pKD4_do_as-bcs2	CCCGAGTATACCCGATCGCAGCGGCATTAAGAGAGG CGCTATCTGAAAACTTACATATGAATATCCTCCTTAGTTC	Used for amplification of the FRT-flanked Km^R cassette from pKD4 with ~50 bp overhangs homologous to the *bcs*-flanking regions
bcs_150bp-up-s	GTCATTGCTGGCATTCTTGC	Used for PCR-verification of *bcs* region replacement by the KmR cassette
KanCst_pKD4_up_as_k1	CAGTCATAGCCGAATAGCCT	Used for PCR-verification of *bcs* region replacement by the KmR cassette

Table S2 | Bacterial strains

Strain	Description / genotype	Source
Escherichia coli		
DH5α	F⁻ λ⁻ *Φ80′ lacZ*ΔM15 Δ(*argF-lac*) U169 *phoA supE44 recA1 relA1 endA1 thi-1 hsdR17*(r_k^-, m_k^+) *gyrA96* | Host strain for general cloning	Lab collection
BL21 Star™ (DE3) Δ*bcs*	F⁻ *ompT hsdS*$_B$ (r_B^-, m_B^-) *gal dcm rne131* (DE3) *bcs* | Host strain for recombinant Bcs complex expression lacking endogenous *bcs* genes	This study
NiCo21(DE3)	*can::CBD fhuA2 [lon] ompT gal (λ DE3) [dcm] arnA::CBD slyD::CBD glmS6Ala* Δ*hsdS λ DE3 = λ sBamHIo* Δ*EcoRI-B int::(lacI::PlacUV5::T7 gene1) i21* Δ*nin5* | Host strain for recombinant protein complex overexpression	New England Biolabs
1094 WT	Wild-type commensal *E. coli* 1094 strain	Jean-Marc Ghigo
1094 Δ*bcsR*	*E. coli* 1094 featuring in-frame non-polar deletion of *bcsR*, chloramphenicol-resistant	Jean-Marc Ghigo

Table S3 | Cryo-EM data collection

**Data collection and Pre-processing**	
Microscope	Titan Krios (CM01 ESRF)
Voltage	300
Camera	Gatan K3
Energy filter	Gatan GIF Quantum LS
Pixel size (Å^2)	0.839
Collection mode	counting with super-resolution
Total electron dose (e$^-$/Å^2)	49.35
Movies	20,022
Defocus range (µm)	-0.3 to -2.1
Motion correction	MotionCor2
CTF correction	Patch CTF (CryoSPARC)
Particle picking	Template picker (CryoSPARC)
Initial model	Ab-Initio (CryoSPARC)
Symmetry	C1

Table S4 | Map and model refinement

Cryo-EM data processing							
**Assembly**	**BcsB 'crown'**	**'crown'-less complex, c-di-GMP-saturated**	**BcsA-B^{TA}-G$^{NTD}_3$ (c-di-GMP-bound)**	**BcsEF (c-di-GMP-bound synthase)**	**'crown'-less complex, c-di-GMP-free BcsA**	**BcsA-B^{TA}-G$^{NTD}_3$ (c-di-GMP-free)**	**BcsEFRQ (c-di-GMP-free synthase)**
**Map refinement**							
Single particles	834,195	260,501	259,200	260,501	314,979	314,979	275,132
Resolution, Å<br>FSC 0.143 | masked / unmasked)	2.35 / 3.2	2.85 / 3.5	3.60 / 4.1	2.85 / 4.1	3.37 / 4	3.43 / 4.2	3.76 / 4.6
Sharpening B-factor	80	89.5	116.3	89.5	105.6	90.2	112.5
**Atomic model refinement**							
# of protein chains / residues	6 / 3,864	15 / 2,895	5 / 1,299	4 / 1,084	13 / 2,877	5 / 1,254	8 / 1,600
# of atoms	30,144	23,702	10,740	8,853	23,441	10,272	12,994
Ligands	-	C2E: 6, ATP: 2, Mg: 2	C2E: 2	C2E: 4	C2E: 4, ATP: 2, Mg: 2	-	C2E: 4, ATP: 2, Mg: 2
B-factors							
Protein (min/max/mean)	11.56/139.94/46.49	18.75/282.17/80.66	27.73/171.53/76.07	24.74/158.55/66.44	0.00/185.64/66.55	0.00/154.56/58.01	26.93/185.64/72.84
Ligand (min/max/mean)	-	20.00/72.85/49.32	44.23/72.85/55.99	30.29/55.45/40.07	35.06/72.85/53.04	-	35.06/72.85/53.04
Bonds							
Length, Å (# > 4σ)	0.002 (0)	0.003 (0)	0.003 (0)	0.004 (0)	0.002 (0)	0.004 (0)	0.002 (0)
Angles, º (# > 4σ)	0.465 (4)	0.491 (0)	0.530 (0)	0.580 (3)	0.465 (7)	0.645 (9)	0.458 (0)
Ramachandran plot							
Favored (%)	97.47	96.84	95.86	96.93	97.74	96.83	97.45
Allowed (%)	2.48	3.09	4.14	3.07	2.19	3.17	2.55
Outliers (%)	0.06	0.07	0.00	0.00	0.07	0.00	0.00
Rotamer outliers (%)	0.84	0.88	0.98	0.21	0.76	0.55	0.87
Cβ outliers (%)	0.00	0.00	0.00	0.00	0.00	0.00	0.00
CaBLAM outliers (%)	1.34	1.35	1.59	1.69	1.29	0.83	1.49
Peptide plane (%)							
Cis proline / general	2.6 / 0.0	0.00 / 0.00	0.0 / 0.0	0.0 / 0.0	0.00	0.0 / 0.0	0.00 / 0.00
Twisted proline / general	0.0 / 0.0	0.00 / 0.00	0.0 / 0.0	0.0 / 0.0	0.00	0.0 / 0.0	0.00 / 0.00
CC (mask)	0.86	0.77	0.62	0.80	0.56	0.72	0.52
Occupancy							
Mean	1.00	1.00	1.00	1.00	1.00	1.00	1.00
occ = 1 (%)	99.93	99.58	100.00	99.80	99.57	100.00	99.23
Clashscore	6.15	7.07	12.89	6.97	8.59	9.56	9.32
Molprobity	1.44	1.58	1.91	1.57	1.52	1.70	1.60

Supplementary References

1. Abidi, W., Torres-Sánchez, L., Siroy, A. & Krasteva, P. V. Weaving of bacterial cellulose by the Bcs secretion systems. *FEMS Microbiology Reviews* **46**, fuab051 (2022).

2. Krasteva, P. V. Bacterial synthase-dependent exopolysaccharide secretion: a focus on cellulose. *Current Opinion in Microbiology* **79**, 102476 (2024).

3. Punjani, A., Rubinstein, J. L., Fleet, D. J. & Brubaker, M. A. cryoSPARC: algorithms for rapid unsupervised cryo-EM structure determination. *Nat Methods* **14**, 290–296 (2017).

4. Abramson, J. *et al.* Accurate structure prediction of biomolecular interactions with AlphaFold 3. *Nature* ***630***, pages 493–500 (2024).

5. Mirdita, M. *et al.* ColabFold: making protein folding accessible to all. *Nat Methods* **19**, 679–682 (2022).

6. Abidi, W., Zouhir, S., Caleechurn, M., Roche, S. & Krasteva, P. V. Architecture and regulation of an enterobacterial cellulose secretion system. *Sci. Adv.* **7**, eabd8049 (2021).

7. Sanchez-Garcia, R. *et al.* DeepEMhancer: a deep learning solution for cryo-EM volume post-processing. *Commun Biol* **4**, 874 (2021).

8. Zheng, S. Q. *et al.* MotionCor2: anisotropic correction of beam-induced motion for improved cryo-electron microscopy. *Nat Methods* **14**, 331–332 (2017).

9. Pettersen, E. F. *et al.* UCSF Chimera: A visualization system for exploratory research and analysis. *J. Comput. Chem.* **25**, 1605–1612 (2004).
