# Supplementary material for: Structural basis for synthase activation and cellulose modification in the E. coli Type II Bcs secretion system
Source: Nat Commun. 2024 Oct 11;15:8799. doi: 10.1038/s41467-024-53113-8 (PMC11470070; doi:10.1038/s41467-024-53113-8)
Supplement: Supplementary file 3 — Reporting Summary [file 41467_2024_53113_MOESM3_ESM.pdf]

nature portfolio

Corresponding author(s): Petya Violinova KRASTEVA

Last updated by author(s): 04/09/2024

Reporting Summary

Nature Portfolio wishes to improve the reproducibility of the work that we publish. This form provides structure for consistency and transparency in reporting. For further information on Nature Portfolio policies, see our Editorial Policies and the Editorial Policy Checklist.

Statistics

For all statistical analyses, confirm that the following items are present in the figure legend, table legend, main text, or Methods section.

n/a	Confirmed	
☐	☒	The exact sample size (*n*) for each experimental group/condition, given as a discrete number and unit of measurement
☒	☐	A statement on whether measurements were taken from distinct samples or whether the same sample was measured repeatedly
☒	☐	The statistical test(s) used AND whether they are one- or two-sided<br>*Only common tests should be described solely by name; describe more complex techniques in the Methods section.*
☒	☐	A description of all covariates tested
☒	☐	A description of any assumptions or corrections, such as tests of normality and adjustment for multiple comparisons
☒	☐	A full description of the statistical parameters including central tendency (e.g. means) or other basic estimates (e.g. regression coefficient) AND variation (e.g. standard deviation) or associated estimates of uncertainty (e.g. confidence intervals)
☒	☐	For null hypothesis testing, the test statistic (e.g. *F*, *t*, *r*) with confidence intervals, effect sizes, degrees of freedom and *P* value noted<br>*Give P values as exact values whenever suitable.*
☒	☐	For Bayesian analysis, information on the choice of priors and Markov chain Monte Carlo settings
☒	☐	For hierarchical and complex designs, identification of the appropriate level for tests and full reporting of outcomes
☒	☐	Estimates of effect sizes (e.g. Cohen's *d*, Pearson's *r*), indicating how they were calculated

Our web collection on statistics for biologists contains articles on many of the points above.

Software and code

Policy information about availability of computer code

Data collection	For structure resolution, cryo-EM data was collected at the CM01 beamline at the European Synchrotron Radiation Facility (ESRF, Grenoble) on a Titan Krios transmission cryo-electron microscope, operated at 300 kV and equipped with a Gatan K3 direct electron detector and a Quantum LS imaging filter.
Data analysis	The movies were motion-corrected using MotionCor2 within the ESRF autoprocessing pipeline and the resulting micrographs were imported in CryoSPARC v4.4.1. Atomic model building and refinements were performed by AlphaFold3 or ColabFold-generated models as inputs for manual building in Coot and automated real-space refinement in Phenix. Interface analyses were carried out with the PISA server.

For manuscripts utilizing custom algorithms or software that are central to the research but not yet described in published literature, software must be made available to editors and reviewers. We strongly encourage code deposition in a community repository (e.g. GitHub). See the Nature Portfolio guidelines for submitting code & software for further information.

Data

Policy information about availability of data

All manuscripts must include a data availability statement. This statement should provide the following information, where applicable:

- Accession codes, unique identifiers, or web links for publicly available datasets
- A description of any restrictions on data availability
- For clinical datasets or third party data, please ensure that the statement adheres to our policy

All data needed to evaluate the conclusions in the paper are present in the paper and/or the Supplementary Information. Refined structural models and electron

density maps are deposited in the electron microscopy and protein databanks with accession codes as follows: EMD-50584 and EMD-50595 for the low-pass filtered global assemblies of the c-di-GMP-saturated and non-saturated Bcs macrocomplex, respectively; 9FMT / EMD-50567 for the locally refined BcsB periplasmic crown; 9FMZ / EMD-50581 and 9FMV / EMD-50571 for the locally refined c-di-GMP-bound and c-di-GMP-free BcsAG3 complex, respectively; 9FNN / EMD-50599 and 9FP0 / EMD-50632 for the locally refined crownless the c-di-GMP-saturated and non-saturated Bcs macrocomplex, respectively; 9FO7 / EMD-50619 for the locally refined BcsE2F2 regulatory subcomplex from the c-di-GMP-saturated state; and 9FP2 / EMD-50633 for the locally refined BcsRQEF vestibule complex from the non-saturated Bcs macrocomplex. Previously published structural models discussed in this work refer to entries 6YB3 (crystal structure of E. coli BcsRQ), 6TJ0 (crystal structure of splayed BcsE), 6YBB (crystal structure in closed BcsE, in a BcsRQ-bound complex), 6PCZ (a BcsGCTD crystal structure), 5FGN (crystal structure of N. meningitidis EptA), 6WLB (cryo-EM structure of poplar CesA8), 4P00 (a crystal structure of R. sphaeroides BcsAB). AlphaFold and ColabFold-generated models used in initial model building or structure analyses are deposited as an open access dataset in Zenodo (DOI:10.5281/zenodo.13732043).

Research involving human participants, their data, or biological material

Policy information about studies with human participants or human data. See also policy information about sex, gender (identity/presentation), and sexual orientation and race, ethnicity and racism.

Reporting on sex and gender	N/A
Reporting on race, ethnicity, or other socially relevant groupings	N/A
Population characteristics	N/A
Recruitment	N/A
Ethics oversight	N/A

Note that full information on the approval of the study protocol must also be provided in the manuscript.

Field-specific reporting

Please select the one below that is the best fit for your research. If you are not sure, read the appropriate sections before making your selection.

[x] Life sciences [ ] Behavioural & social sciences [ ] Ecological, evolutionary & environmental sciences

For a reference copy of the document with all sections, see nature.com/documents/nr-reporting-summary-flat.pdf

Life sciences study design

All studies must disclose on these points even when the disclosure is negative.

Sample size	No statistical methods were used to predetermine sample size (included in the Methods statement). Minimum three independent experiments for phenotypic and biochemical data. Loading or positive/negative controls included in each experiment. Cryo-EM data collection on 2 grids with merged data for increased throughput within the allocated synchrotron collection time (48 hours).
Data exclusions	None as relevant to the effects/mutants reported in the manuscript. Through the course of the study additional mutants and expression construct were generated and tested, however they are not key to, are not discussed in and do not contradict in any way the represented data and conclusions.<br><br>Regarding cryo-EM data processing, standard data clean-up procedures were carried out, such as removal of micrographs outside the desired defocus and resolution ranges and removal of particles from 2D or 3D classes with unresolved structural features (processing details provided in the Methods section).
Replication	Yes, described in figure legends.
Randomization	None (included in Methods statement).
Blinding	None (included in Methods statement). However some experiments, such as phenotypic assays of cellulose secretion, were either reproduced by different researchers or had multiple researchers carry different steps of the respective protocols.

Behavioural & social sciences study design

All studies must disclose on these points even when the disclosure is negative.

Study description	N/A
Research sample	N/A
Sampling strategy	N/A

Data collection	N/A
Timing	N/A
Data exclusions	N/A
Non-participation	N/A
Randomization	N/A

Ecological, evolutionary & environmental sciences study design

All studies must disclose on these points even when the disclosure is negative.

Study description	N/A
Research sample	N/A
Sampling strategy	N/A
Data collection	N/A
Timing and spatial scale	N/A
Data exclusions	N/A
Reproducibility	N/A
Randomization	N/A
Blinding	N/A

Did the study involve field work? ☐ Yes ☒ No

Field work, collection and transport

Field conditions	N/A
Location	N/A
Access & import/export	N/A
Disturbance	N/A

Reporting for specific materials, systems and methods

We require information from authors about some types of materials, experimental systems and methods used in many studies. Here, indicate whether each material, system or method listed is relevant to your study. If you are not sure if a list item applies to your research, read the appropriate section before selecting a response.

Materials & experimental systems

n/a	Involved in the study	
☐	☒	Antibodies
☒	☐	Eukaryotic cell lines
☒	☐	Palaeontology and archaeology
☒	☐	Animals and other organisms
☒	☐	Clinical data
☒	☐	Dual use research of concern
☒	☐	Plants

Methods

n/a	Involved in the study	
☒	☐	ChIP-seq
☒	☐	Flow cytometry
☒	☐	MRI-based neuroimaging

Antibodies

Antibodies used	Commercial antibodies with catalog numbers and concentrations provided in the manuscript as follows: Mouse anti-HA (hemagglutinin) (Thermo Fisher Scientific, #26183; dilution 1:1000) and mouse anti-STREP II (QIAGEN, #34850; dilution 1:1000) antibodies were used as primary antibodies; horseradish peroxidase (HRP)–conjugated rabbit anti-mouse antibody (Abcam, ab6728; dilution 1:10,000) was used as secondary antibody
Validation	Mouse anti-HA antibody (Thermo Fisher Scientific, #26183) validation and specification can be found at manufacturer's webpage: https://www.thermofisher.com/antibody /product/HA-Tag-Antibody-clone-2-2-2-14-Monoclonal/26183<br><br>Mouse anti-STREP II antibody (QIAGEN, #34850) validation and specification can be found at manufacturer's webpage: https://www.qiagen.com/us/products/discovery-and-translational-research/protein-purification/tagged-protein-expression-purification-detection/strep-tag-antibody<br><br>Horseradish peroxidase (HRP)–conjugated rabbit anti-mouse antibody (Abcam, ab6728) validation and specification can be found at manufacturer's webpage: https://www.abcam.com/en-fr/products/secondary-antibodies/rabbit-mouse-igg-h-l-hrp-ab6728

Eukaryotic cell lines

Policy information about cell lines and Sex and Gender in Research

Cell line source(s)	N/A
Authentication	N/A
Mycoplasma contamination	N/A
Commonly misidentified lines (See ICLAC register)	N/A

Palaeontology and Archaeology

Specimen provenance	N/A
Specimen deposition	N/A
Dating methods	N/A

☐ Tick this box to confirm that the raw and calibrated dates are available in the paper or in Supplementary Information.

Ethics oversight	N/A

Note that full information on the approval of the study protocol must also be provided in the manuscript.

Animals and other research organisms

Policy information about studies involving animals; ARRIVE guidelines recommended for reporting animal research, and Sex and Gender in Research

Laboratory animals	N/A
Wild animals	N/A
Reporting on sex	N/A
Field-collected samples	N/A
Ethics oversight	N/A

Note that full information on the approval of the study protocol must also be provided in the manuscript.

Clinical data

Policy information about clinical studies

All manuscripts should comply with the ICMJE guidelines for publication of clinical research and a completed CONSORT checklist must be included with all submissions.

Clinical trial registration	N/A
Study protocol	N/A
Data collection	N/A
Outcomes	N/A

Dual use research of concern

Policy information about dual use research of concern

Hazards

Could the accidental, deliberate or reckless misuse of agents or technologies generated in the work, or the application of information presented in the manuscript, pose a threat to:

No	Yes	
☒	☐	Public health
☒	☐	National security
☒	☐	Crops and/or livestock
☒	☐	Ecosystems
☒	☐	Any other significant area

Experiments of concern

Does the work involve any of these experiments of concern:

No	Yes	
☒	☐	Demonstrate how to render a vaccine ineffective
☒	☐	Confer resistance to therapeutically useful antibiotics or antiviral agents
☒	☐	Enhance the virulence of a pathogen or render a nonpathogen virulent
☒	☐	Increase transmissibility of a pathogen
☒	☐	Alter the host range of a pathogen
☒	☐	Enable evasion of diagnostic/detection modalities
☒	☐	Enable the weaponization of a biological agent or toxin
☒	☐	Any other potentially harmful combination of experiments and agents

Plants

Seed stocks	N/A
Novel plant genotypes	N/A
Authentication	N/A

ChIP-seq

Data deposition

☐ Confirm that both raw and final processed data have been deposited in a public database such as GEO.

☐ Confirm that you have deposited or provided access to graph files (e.g. BED files) for the called peaks.

Data access links<br>*May remain private before publication.*	N/A
Files in database submission	N/A
Genome browser session<br>(e.g. UCSC)	N/A

Methodology

Replicates	N/A
Sequencing depth	N/A
Antibodies	N/A
Peak calling parameters	N/A
Data quality	N/A
Software	N/A

Flow Cytometry

Plots

Confirm that:

☐ The axis labels state the marker and fluorochrome used (e.g. CD4-FITC).

☐ The axis scales are clearly visible. Include numbers along axes only for bottom left plot of group (a 'group' is an analysis of identical markers).

☐ All plots are contour plots with outliers or pseudocolor plots.

☐ A numerical value for number of cells or percentage (with statistics) is provided.

Methodology

Sample preparation	N/A
Instrument	N/A
Software	N/A
Cell population abundance	N/A
Gating strategy	N/A

☐ Tick this box to confirm that a figure exemplifying the gating strategy is provided in the Supplementary Information.

Magnetic resonance imaging

Experimental design

Design type	N/A
Design specifications	N/A
Behavioral performance measures	N/A

Acquisition

Imaging type(s)	N/A
Field strength	N/A
Sequence & imaging parameters	N/A
Area of acquisition	N/A

Diffusion MRI ☐ Used ☐ Not used

Preprocessing

Preprocessing software	N/A
Normalization	N/A
Normalization template	N/A
Noise and artifact removal	N/A
Volume censoring	N/A

Statistical modeling & inference

Model type and settings	N/A
Effect(s) tested	N/A

Specify type of analysis: ☐ Whole brain ☐ ROI-based ☐ Both

Statistic type for inference (See Eklund et al. 2016)	N/A
Correction	N/A

Models & analysis

n/a	Involved in the study
☐	☐ Functional and/or effective connectivity
☐	☐ Graph analysis
☐	☐ Multivariate modeling or predictive analysis

Functional and/or effective connectivity	N/A
Graph analysis	N/A
Multivariate modeling and predictive analysis	N/A
